# Effects of wearing surgical masks on fraction of inspired oxygen in spontaneously breathing patients: improving safety for frontline healthcare professionals under pandemic situations

**DOI:** 10.1186/s12871-022-01649-x

**Published:** 2022-04-18

**Authors:** Kazuhiro Minoguchi, Akira Isii, Toshiki Nakamura, Hitoshi Sato, Takeru Abe, Hiromasa Kawakami, Kyota Nakamura, Takahisa Goto

**Affiliations:** 1 Department of Anesthesiology, Yokohama City University Medical Center, 4-57 Urafunecho, Minami-ku, Yokohama, Kanagawa 232-0024 Japan; 2 Department of Clinical Quality Management, Osaka University Hospital, 2-15 Yamadaoka, Suita, Osaka 565-0871 Japan; 3 Department of Quality and Safety in Healthcare, Yokohama City University Medical Center, 4-57 Urafunecho, Minami-ku, Yokohama, Kanagawa 232-0024 Japan; 4 Department of Anesthesiology and Intensive Care Unit, Yokohama City University Graduate School of Medicine, 3-9 Fukuura, Kanazawa-ku, Yokohama, Kanagawa 236-0004 Japan

**Keywords:** COVID-19, Fraction of inspired oxygen, Oxygen mask, Respiratory infections, Surgical mask, Preventing infection

## Abstract

**Background:**

During pandemic situations, many guidelines recommend that surgical masks be worn by both healthcare professionals and infected patients in healthcare settings. The purpose of this study was to clarify the levels and changes of oxygen concentration over time while oxygen was administered over a surgical mask.

**Methods:**

Patients scheduled to undergo general anesthesia (*n* = 99) were enrolled in this study. First, patients were administered oxygen at 6 L/min via an oxygen mask over a surgical mask for 5 min. The patients removed the surgical mask and then took a 3-min break; thereafter, the same amount of oxygen was administered for another 5 min via the oxygen mask. We measured the fraction of inspired oxygen (FiO_2_), the end-tidal CO_2_ (EtCO_2_), and respiratory frequency every minute for 5 min, both while administering oxygen with and without a surgical mask. The FiO_2_ was measured at the beginning of inspiration and the EtCO_2_ was measured at the end of expiration.

**Results:**

The FiO_2_ at 5 min was significantly lower when breathing with a surgical mask than that without it (mean difference: 0.08 [95% CI: 0.067–0.10]; *p* <  0.001). In contrast, the EtCO_2_ at 5 min was significantly higher when breathing with a surgical mask than that without it (mean difference: 11.9 mmHg [95% CI: 10.9–12.9]; *p* <  0.001).

**Conclusion:**

The FiO_2_ was lower when oxygen was administered over surgical masks than when patients did not wear surgical masks. Oxygen flow may need to be adjusted in moderately ill patients requiring oxygen administration.

## Background

The coronavirus disease 2019 (COVID-19) pandemic caused by the novel severe acute respiratory syndrome coronavirus 2 (SARS-CoV-2) has cumulatively infected 462.7 million people globally and resulted in 6.0 million deaths as of March 2022 [1].

The main route of infection for SARS-CoV-2 is through respiratory droplets during close contact (direct physical or face-to-face contact with a probable or confirmed case) for prolonged periods of time [2]. This indicates that wearing face masks and maintaining social distance are effective preventive measures against community spread of infections. A study conducted in New York City to determine whether requesting citizens to wear face masks could reduce infection showed that the number of new COVID-19 infections and deaths per day decreased after the request was issued [3]. Although there are several variables that make it difficult to obtain reliable results in community settings, many other studies have found that the use of face masks or a combination of face masks and social distancing are useful in preventing infection spread [4–9]. Similarly, the infection-preventive effect of face masks (especially non-woven surgical masks) in healthcare settings has been established [10].

Patients with severe respiratory infections may require various oxygen therapies, thereby increasing the risk of infection to healthcare professionals [11]. Tracheal intubation and extubation are procedures that are most likely to produce large amounts of aerosols; additionally coughing in spontaneously breathing patients is believed to produce a comparable amount of aerosol [12–14]. As the function of the surgical masks is to prevent the spread of droplets—excreted by infected patients due to coughing or other methods—to the surrounding environment, many guidelines recommend that surgical masks be worn not only by healthcare professionals but also by infected patients in a healthcare setting [15–18]. It is thought that the risk of infection to healthcare professionals can be reduced if oxygen is administered over a surgical mask, although there is no strong evidence. Recently, a small study on healthy volunteers was published to address this question [19], but there are no reports that clarify the level of oxygen concentration that needs to be maintained in patients in clinical situations.

The purpose of this study was to clarify the level and changes of oxygen concentration while oxygen was administered over a surgical mask over time.

## Methods

This prospective observational study was conducted at the Yokohama City University Medical Center (Minami-ku, Yokohama, Japan) between January and March 2021. This study was approved by the Yokohama City University Certified Institutional Review Board (B201000011), and written informed consent was obtained from all subjects prior to general anesthesia. This study was registered with the UMIN-CTR on December 15, 2020 (Registry number: UMIN000042751).

### Study protocol

**Fig. 1:**
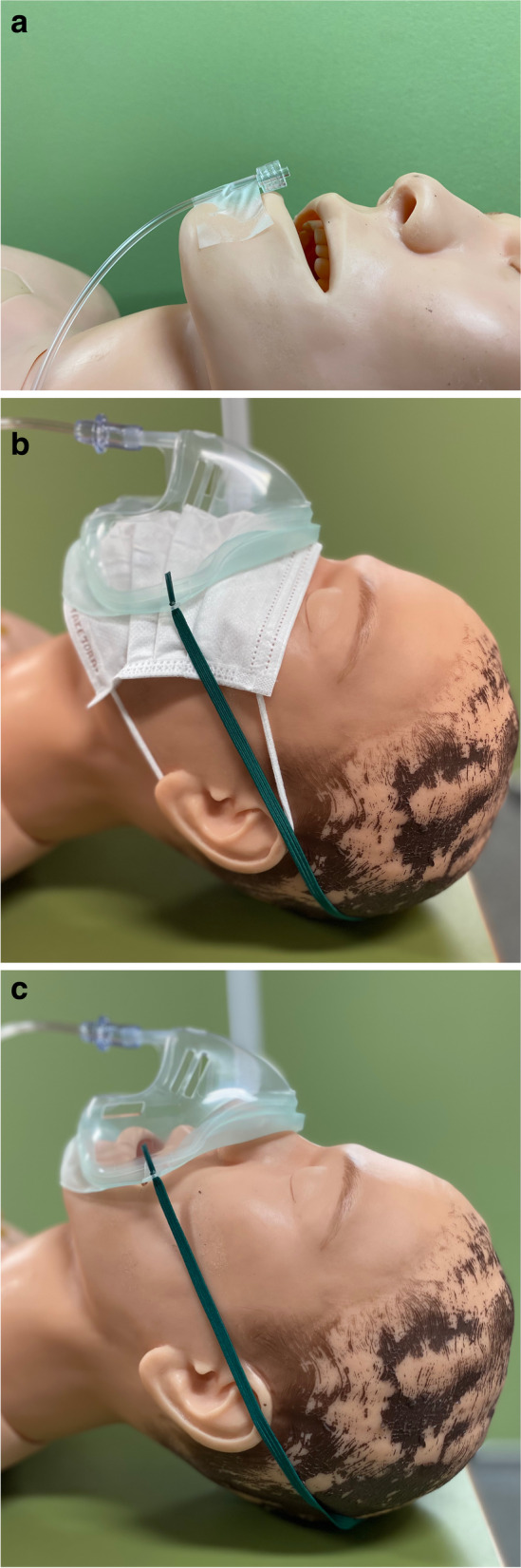
**a** Sampling tube location Ⓒ2021 Hitoshi Sato. No Rights Reserved. **b** Oxygen mask with a surgical mask Ⓒ2021 Hitoshi Sato. No Rights Reserved. **c** Oxygen mask only Ⓒ2021 Hitoshi Sato. No Rights Reserved

Adult patients with an American Society of Anesthesiologists physical status of I–II who underwent general anesthesia for elective surgery were enrolled in this study. Patients with respiratory complications were excluded from this study. Baseline characteristics of patients, such as sex, age, height, and weight, were obtained. Patients were asked to put on a uniform non-woven surgical mask at the entrance of the operation theater. After entering the operating theater, they were placed in a supine position on the operating table and were attached to standard monitoring devices for continuous electrocardiogram, non-invasive blood pressure, and oxygen saturation (using pulse oximetry). Prior to induction of general anesthesia, the tip of the gas sampling tube was taped over the lower lip and connected to an anesthetic gas module (Philips IntelliVue G5-M1019A, Philips Electronics NV, Amsterdam, Netherlands) to monitor respiratory gases (Fig. 1a).

The patients were instructed to breathe normally, next, an oxygen mask *(*EcoLite*;* Intersurgical*,* Wokingham, UK) was attached over the surgical mask, and the oxygen flow rate was set to 6 L/min (Fig. 1b). We measured the fraction of inspired oxygen (FiO_2_), the end-tidal CO_2_ (EtCO_2_), and respiratory frequency every minute for 5 min (as in our pilot study, it took 5 min for the oxygen concentration to reach a steady state) [20]. No conversation was made during the measurement, and the rest of the environment was maintained stable. No specific instructions were given as to whether to breathe through the nose or mouth, because individuals do not consciously make this distinction during normal breathing. The patients were asked to remove the surgical mask after 5 min and were given a 3-min break (Fig. 1c). After the break, the FiO_2_, the EtCO_2_, and respiratory frequency were measured at the same oxygen flow rate via oxygen mask every minute for another 5 min. After the measurements were completed, the sampling tube was removed, and general anesthesia was induced. The FiO_2_ was measured at the beginning of inspiration and the EtCO_2_ was measured at the end of expiration.

The primary outcome of this study was the difference in the FiO_2_ between patients with and without a surgical mask after 5 min of O_2_ delivery. The main secondary outcome was the difference in the EtCO_2_ under the same conditions.

### Sample size

Sample size was calculated assuming a power of 90% and α = 0.05 to perform paired t-test for analysis of the fraction of inspired oxygen. Based on our pilot study, we obtained an absolute difference of 0.05 in oxygen level between subjects with (0.60 ± 0.11, mean ± standard deviation [SD]) and without (0.65 ± 0.09: mean ± SD) masks [20]. The estimated total sample size was calculated to be 95 patients using PASS 14 Power Analysis and Sample Size Software (NCSS, Kaysville, UT), while considering an exclusion rate of 5% owing to potentially incomplete data.

### Statistical analysis

The paired t-test was performed to analyze the FiO_2_, the EtCO_2_, and the SpO_2_ after 5 min of O_2_ delivery. A two-sided *p*-value < 0.05 was considered as statistically significant.

Repeated measures analysis of variance (ANOVA) was applied to the FiO_2_, the EtCO_2_, and respiratory rate to evaluate within-subjects (time) and between-subjects (mask) effects. The Bonferroni correction was used to account for multiple comparisons. Statistical analyses were performed using SPSS Statistics for Windows (version 25.0; IBM, Armonk, NY).

## Results

In total, 99 subjects were enrolled. The baseline characteristics of the enrolled participants are shown in Table 1. The mean age of all subjects was 58.4 years, and 66% of all subjects were female.

**Table 1:** Patients’ characteristics

n	99
Sex (M/F)	33/66
Mean age, years	58.4 ± 17.6
Mean height, cm	167.6 ± 7.0/154.9 ± 6.6
Mean weight, kg (M/F)	68.4 ± 10.4/54.1 ± 9.7
ASA physical status I/II	19/80
Mean baseline SpO_2_, %	98.9 ± 1.5

Data are presented as mean ± standard deviation

*M* male, *F* female, *ASA* American Society of Anesthesiologists, *SD* standard deviation

The FiO_2_ at 5 min was significantly lower when breathing with a surgical mask than that without it (mean difference: 0.08 [95% CI: 0.067–0.10]; *p* <  0.001).

There was no significant difference in the FiO_2_ with a surgical mask between 4 and 5 min (mean difference: 0.0068 [95% CI: − 0.002 to 0.015; *p* = 1.000]). There was also no significant difference in the FiO_2_ without a surgical mask at 4 and 5 min (mean difference: 0.01 [95% CI: 0.003–0.02; *p* = 0.095]). Both groups were considered to have reached a steady state within 5 min. The EtCO_2_ at 5 min was significantly higher while breathing with a surgical mask than that without it (mean difference: 11.9 mmHg [95% CI: 10.9–12.9]; *p* <  0.001).

**Fig. 2:**
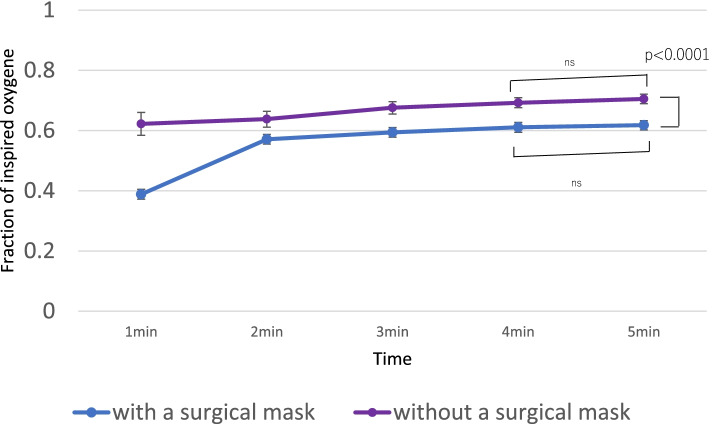
Fraction of inspired oxygen at each timepoint. Error bars are 95% CIs. CI, confidence interval

**Fig. 3:**
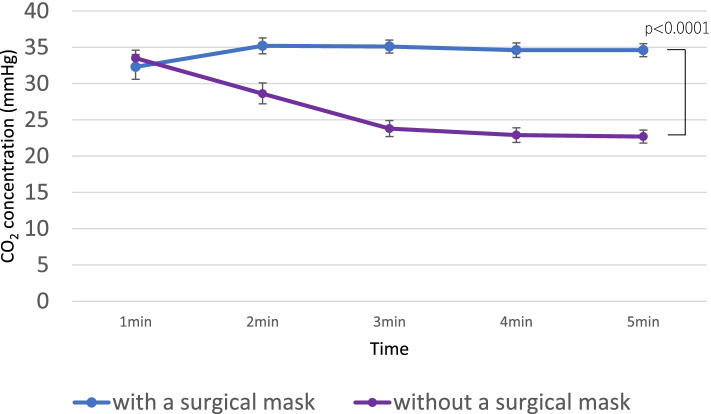
End-tidal CO_2_ at each timepoint. Error bars are 95% CIs. CI, confidence interval

**Fig. 4:**
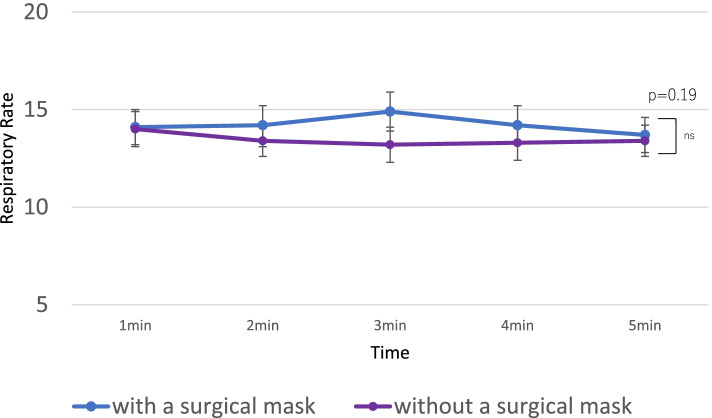
Respiratory rate at each timepoint. Error bars are 95% CIs. CI, confidence interval. RR, respiratory rate

There was no significant difference in respiratory rate during breathing with and without a surgical mask (*p* = 0.19) (Figs. 2, 3 and 4, Tables 2 and 3).

**Table 2:** Results of F_i_O_2_, EtCO_2_, and RR with or without a surgical mask

	Mean (SD)		Mean difference (95% CI)	P
	With a mask	Without a mask		
FiO_2_	0.56 (0.15)	0.67 (0.08)	0.11 (0.095–0.125)	< 0.0001
EtCO_2_	34.4 (5.9)	26.3 (7.0)	8.1 (7.2–8.9)	< 0.0001
RR	13.7 (4.4)	13.4 (4.1)	0.31(−1.52 ~ 0.89)	0.19

*P-*values from repeated measures analysis of variance (ANOVA)

*FiO*_*2*_ fraction of inspired oxygen, *EtCO*_*2*_ end-tidal CO_2_, *SD* standard deviation, *CI* confidence interval, *RR* respiratory rate

**Table 3:** Trend over time of FiO_2_, EtCO_2_, and RR

	With a surgical mask: Mean (SD)	Without a surgical mask: Mean (SD)	*P* value
**FiO**_**2**_			
1 min	0.39 (0.19)	0.62 (0.084)	< 0.0001
2 min	0.57 (0.13)	0.64 (0.080)	< 0.0001
3 min	0.59 (0.10)	0.68 (0.082)	< 0.0001
4 min	0.61 (0.085)	0.69 (0.083)	< 0.0001
5 min	0.62 (0.081)	0.71 (0.077)	< 0.0001
**EtCO**_**2**_**(mmHg)**			
1 min	32.3 (8.52)	33.5 (5.50)	0.1954
2 min	35.2 (5.48)	28.6 (7.23)	< 0.0001
3 min	35.1 (4.60)	23.8 (5.56)	< 0.0001
4 min	34.6 (5.03)	22.9 (5.05)	< 0.0001
5 min	34.6 (4.37)	22.7 (4.45)	< 0.0001
**RR**			
	With a surgical mask: Mean (SD)	Without a surgical mask: Mean (SD) (Standard Deviation)	*P* value
1 min	14.1 (4.6)	14.0 (4.3)	0.75
2 min	14.2 (5.3)	13.4 (4.4)	0.076
3 min	14.9 (5.1)	13.1 (4.7)	< 0.0001
4 min	14.2 (4.8)	13.3 (4.3)	0.0024
5 min	13.7 (4.4)	13.4 (4.1)	0.30

*FiO*_*2*_ fraction of inspired oxygen, *EtCO*_*2*_ end-tidal CO_2_, *SD* standard deviation, *RR* respiratory rate

There was no significant difference in the SpO_2_ at 5 min with and without a surgical mask (99.9 ± 0.4% vs 99.9 ± 0.2, *p* = 0.29).

## Discussion

This study focused on whether oxygen can be safely administered over a surgical mask. The study results show that when patients wore surgical masks and oxygen was administered, the FiO_2_ was lower than when patients did not wear surgical masks. This finding may be important in moderately ill patients that require oxygen administration. This is because if the FiO_2_ under surgical mask use is approximately 8–10% lower than what is conventionally expected, increased oxygen flow rates may be needed to be considered in some cases. Recently, a study in healthy volunteers showed that oxygen administered through a surgical mask may result in a lower inhaled oxygen concentration, and oxygen flow rates of 5 and 10 L/min showed similar trends [19]. Considering this evidence, we postulated that the measurement of our study parameters with an oxygen flow rate of 6 L/min would be a good reflection of the influence of the surgical mask.

Furthermore, the FiO_2_ and the EtCO_2_ were measured using a sampling tube taped on the lower lip in our study. In a previous study, the oxygen concentration at the mouth with normal oxygen administration was correlated with the oxygen concentration in the pharynx, and the concentration at the pharynx was found to be lower than that in the mouth [21]. If the oxygen concentration at the mouth is low with the use of a surgical mask, the oxygen concentration of the inhalant gas reaching the trachea is expected to be even lower. In this sense, our measured oxygen concentration was not a true FiO_2_ measurement. However, according to the previous study, it can be considered as a good substitution for FiO_2_ under the conditions of our study [21].

Respiratory frequency has also been shown to affect the FiO_2_ during oxygen administration by the oxygen mask [22]. In our study design, measurements were taken in relatively healthy, respiratory complication-free patients in an anesthesia-free situation, and there were no significant differences in respiratory rates with or without surgical masks, which were considered appropriate conditions for comparing oxygen levels.

A possible reason for the decrease in oxygen concentration with a surgical mask was the difference in CO_2_ concentration. That the use of a surgical mask may have resulted in an increased CO_2_ concentration could not be ruled out, and consequently, this may have decreased the FiO_2_. Furthermore, if only an oxygen mask was used, the CO_2_ in the exhaled gas should be diluted by the supplied oxygen, resulting in a low CO_2_ concentration. In fact, it is well known that the oxygen mask contains a mixture of administered oxygen, ambient air, and the patient’s exhaled air during the various phases of breathing. It is imagined that the dilution effect of CO_2_ due to this is reduced by the restriction of airflow with the surgical mask. The difference in the EtCO_2_ in our study was presumably a reflection of these.

Another possible reason for the decrease in oxygen concentration with a surgical mask was the restriction of oxygen flow by the surgical mask. While this reason is possible, our results alone could not explain this. Studies in healthy adults have shown that face masks restrict airflow, which increases breathing resistance and affects respiratory function [23–26]. When the 6-min walk test was performed with a face mask, a prominent increase in respiratory distress owing to airflow obstruction was reported [27]. These studies show that during exercise, the surgical mask interferes with the smooth inflow and outflow of the air. It would be necessary to visualize and examine the airflow around the surgical mask in a resting state.

In addition, the effect of respiratory effort and surgical masks is a matter of concern. Our results show that the use of surgical masks may increase the concentration of CO_2_ in the inspiratory gas. In a previous study, increased PaCO_2_ was shown to increase chemoreceptor-mediated respiratory drive even in healthy participants [28]. In patients with moderate to severe respiratory failure by SARS-CoV-2, intense respiratory drive could cause self-induced lung injury, and our results suggest that the administration of oxygen with a surgical mask in critically ill patients may be potentially harmful.

Finally, our study has some limitations. First, although this study was conducted using only one type of surgical mask and one type of oxygen mask, various other combinations are possible. Oxygen masks have a variety of shapes and characteristics, and the results may differ depending on the type of oxygen mask used. Similarly, the relationship between the size of the oxygen mask and surgical mask may also affect the results. A relatively smaller surgical mask may facilitate the uptake of oxygen into the surgical mask, which may affect the results.

Second, typical severe SARS-CoV-2 patients have severe respiratory distress, anxiety, high respiratory rate, and low compliance with wearing a mask. As our participants were healthy and had a normal respiratory rate, the results of this study cannot be directly applied to patients with severe SARS-CoV-2.

Third, we could not collect arterial blood gas data because our participants were healthy preanesthetic patients. As there was no difference in SpO_2_ between the two groups, our study does not clarify the effect of the surgical mask on partial pressure of oxygen in arterial blood. The same applies to the partial pressure of arterial blood CO_2_. Therefore, to address these limitations, further research is needed.

Furthermore, we had set a 3-min interval between measurements in our study. Previous studies investigating the function of different oxygen masks had set an interval of 90 s. Another study investigating preoxygenation showed that after 5 min of inhalation of 100% oxygen with anesthetic circuit, the partial pressure of arterial blood oxygen returned to baseline within 3 min in room air [29]. We set the interval at 3 min on the basis of these findings. However, it cannot be completely ruled out that the first measurement may have influenced the second, and this may be a limitation of our study.

## Conclusion

We measured the FiO_2_ using a sampling tube taped on the lower lip in our study. In this situation, the FiO_2_ is lower with the use of a surgical mask while administering oxygen than that without a surgical mask. This needs to be considered in moderately ill patients when determining oxygen flow.

## Data Availability

The datasets used and/or analyzed during this study are available from the corresponding author on reasonable request.

## Abbreviations

COVID-19: Coronavirus disease 2019
SARS-CoV-2: novel severe acute respiratory syndrome coronavirus 2
FiO_2_: Fraction of inspired oxygen
EtCO_2_: end-tidal CO_2_
CI: Confidence interval
RR: Respiratory rate
